# Improvements in pincer nail deformity occur earlier and last longer when acetylcysteine gel is added to an overcurvature‐correcting device: Results of a phase 3, multicenter, randomized, vehicle‐controlled, investigator‐blinded study

**DOI:** 10.1111/1346-8138.17010

**Published:** 2023-11-27

**Authors:** Masataka Saito, Akihiko Ikoma, Akira Fujikawa, Kazuaki Tanaka

**Affiliations:** ^1^ Medical Corporation Shinanokai Shinanozaka Clinic Shinjuku‐ku Tokyo Japan; ^2^ Department of Dermatology Keio University School of Medicine Shinjuku‐ku, Tokyo Japan; ^3^ Maruho Co., Ltd. Kita‐ku Osaka Japan; ^4^ Maruho Co., Ltd. Shimogyo‐ku Kyoto Japan

**Keywords:** acetylcysteine (AC) gel, overcurvature‐correcting device, pincer nail, randomized controlled trial, vehicle‐controlled

## Abstract

Acetylcysteine (AC) destabilizes keratin and softens nails, reducing the time needed to correct pincer nail deformity with an overcurvature‐correcting device. The objective of this phase 3, multicenter, randomized, investigator‐blinded study was to evaluate the early and sustained therapeutic effectiveness and safety of 10% AC gel plus an overcurvature‐correcting device to treat pincer nails. Patients aged 12 years and older with hallux pincer nail were fitted with an overcurvature‐correcting device for 7 days, with a 24‐h application of AC gel (*n* = 40) or vehicle (*n* = 39) on day 1. The primary end point (achievement of a distal narrowed nail width ratio ≥70% on day 8) was met by 47.5% in the 10% AC group and 25.6% in the vehicle group (difference 21.9%; *p* = 0.0439). Secondary end points showed a greater tendency towards improvement with 10% AC. The nail correction effect was maintained for at least 12 weeks in the majority of AC‐treated patients, although the study duration was insufficient to assess the long‐term probability of recurrence. No AC‐related adverse events were reported. In conclusion, a single application of 10% AC gel combined with short‐term device use facilitated earlier correction of pincer nails compared with the device alone, with improvements maintained after device removal.

## 1 INTRODUCTION

Pincer nail, a common condition affecting nearly one of 100 individuals worldwide,^1^ generally presents on the hallux toe with both edges of the nail plate excessively curved inwards.^2^ Clinically, pincer nails may cause symptoms ranging from mild discomfort to intolerable pain,^3^, ^4^ which reduces the ability of affected individuals to undertake the usual activities of daily life, including wearing shoes and walking.^1^, ^3^, ^5^

There is no standard treatment for pincer nails, although conservative and surgical procedures can be performed.^1^ Conservative treatment involves attaching an overcurvature‐correcting device to relieve subjective symptoms and revert the transverse overcurvature to as near normal as possible.^6^ Such procedures can take several months to correct the deformity,^2^, ^7^ and for particularly thick nails, simultaneous attachment of two wires, or repeat procedures, may be necessary.^8^ In severe cases, invasive surgery may be required.^2^, ^9^ However, conservative treatment should be considered first, to avoid the risk of permanent deformity caused by surgical treatment, despite the possibility of recurrence or the necessity for additional corrective treatments in patients with pincer nail associated with underlying bone deformity or exostosis.

It has been reported that use of an overcurvature‐correcting device combined with thioglycolic acid, which has a nail‐softening effect, could correct overcurvature within a shorter duration.^7^ Such combination treatments offer the possibility of more rapid correction compared with a device alone, as well as the ability to correct pincer nails that are thick, hard, and intractable. Thus, we developed a nail‐softening agent containing acetylcysteine (AC) as an active ingredient; the thiol group of AC is able to cleave the disulfide bonds within keratin,^10^, ^11^ and a topical gel containing AC can destabilize and soften the nail structure. In a phase 1/2 study, a single 24‐h application of AC gel (either 10%, *n* = 18; 20%, *n* = 18; or 30%, *n* = 17) combined with use of an overcurvature‐correcting device use improved pincer nails at an earlier stage compared with the device alone^12^ (vehicle control; *n* = 17). We report the results from a phase 3 study evaluating the early and sustained therapeutic effectiveness and safety of combination treatment with 10% AC gel and an overcurvature‐correcting device to treat pincer nails.

## 2 METHODS

### 2.1 Study design

This was a phase 3, multicenter, randomized, vehicle‐controlled, investigator‐blinded study in patients with hallux pincer nail. The study design was conceived based on the findings from the investigator‐initiated clinical research conducted by M. Saito et al. (unpublished). The study (jrct2031200264) was conducted at six Japanese facilities (Text S1) between October 2020 and July 2021, in compliance with Good Clinical Practice ordinance, the ethical principles based on the Declaration of Helsinki, and all other legal and regulatory requirements. The institutional review board of each investigational site approved the trial protocol, and all patients provided written informed consent.

On the day of treatment initiation, after the investigator confirmed eligibility, patients were randomly assigned 1:1 using a web enrollment system to receive either 10% AC gel or vehicle. Since it was considered difficult to ensure complete blinding due to the characteristics of the AC gel formulation, the medical personnel responsible for drug allocation and application and the patients themselves were not blinded, but the investigator, all other study personnel, and the sponsor remained blinded until the end of the study.

### 2.2 Patients

Eligible patients were aged 12 years and older at the time of informed consent. The pincer nail could be on either hallux, and was required to have a distal narrowed nail width (dNNW) ratio^13^ of ≥20% and ≤50%. The dNNW ratio was calculated using the formula B/A × 100% (Figure S1), where A was the nail width (measured on day 1, visit 1), and B was the width at the tip (measured at each visit). If the nails of both halluces met the inclusion criteria, the nail with the smaller dNNW ratio was selected for correction.

The main exclusion criteria were patients complicated with ingrown nail, tinea unguium, nail psoriasis, onychogryphosis, or pachyonychia on the pincer nail to be evaluated; patients with a pincer nail plate <1 mm in thickness; and patients with brittle nails, i.e., at increased risk of cracks or nail damage.

### 2.3 Treatment administration

There were four possible treatment periods through which patients could pass (Figure S2), with transitions determined by the dNNW ratio at each stage. All patients completed treatment period 1, and the possible pathways were 1 > 4, 1 > 2 > 4, and 1 > 2 > 3. The study was complete after all assessments were conducted at either visit 11 (period 3) or visit 18 (period 4).

Treatment period 1: on day 1 (visit 1), all eligible patients were fitted with the nail overcurvature‐correcting device (Makizume Meister; Maruho Hatsujyo Kogyo Co., Ltd.) by the investigator. The skin around the nail was then protectively masked (Figure S3), approximately 0.5 g of 10% AC gel or vehicle was applied to the entire pincer nail plate for a period of 24 h, and dressings applied. On day 2 (visit 2), dressings and topical treatment were removed, and on day 8 (visit 3) the device was removed. Patients with a dNNW ratio <70% moved to treatment period 2, and those with a ratio ≥70% moved directly to treatment period 4.
Treatment period 2: the device was reattached on day 8 without further application of the investigational drug. On days 18 (visit 4) and 29 (visit 5), the dNNW ratio was calculated. Patients who continued to have a ratio of <70% on day 29 continued with device attachment and transitioned to treatment period 3. Patients with a ratio of ≥70% on day 18 or day 29 transitioned to treatment period 4.
Treatment period 3: the device remained attached for up to 12 weeks, with dNNW ratios calculated at each visit (visits 6–11, every 2 weeks). As soon as the ratio reached ≥70%, the device was removed, with follow‐up continuing up to visit 11.
Treatment period 4: this period aimed to assess the duration of maintenance of overcurvature correction. At the start of this period, the device was not attached. The patient visited the study site after 1 week (visit 12), 2 weeks (visit 13), and every 2 weeks thereafter, up to 12 weeks (visits 14–18). At each visit, the dNNW ratio was calculated; if it reduced to ≤50% at any time up to week 10, the device was reattached until the ratio reached ≥70% or until week 12.

### 2.4 Study end points

The primary efficacy end point was the percentage of patients who reached the dNNW ratio of 70% on day 8 (visit 3). Secondary end points were the dNNW ratio and the amount of change in the ratio on day 2 and day 8 (or the discontinuation visit). Another secondary end point (for patients who transitioned directly between treatment periods 1 > 4) was the rate of relapse (dNNW ratio ≤ 50%) by visit 18 and the number of days until the ratio became ≤50%. We also explored the dNNW ratio during treatment period 1 according to nail plate thickness (<1.4 mm and ≥1.4 mm).

Safety was evaluated using adverse event (AE) reporting. Events were assessed for severity and for the relationship to the investigational drug or the device. AEs of special interest (AESIs) were inflammation around the nail to which the topical treatment was applied or detachment of the overcurvature‐correcting device.

### 2.5 Statistical analysis

Based on the results of the prior phase 1/2 study,^12^ it was anticipated that 60% of 10% AC‐treated patients and 20% of vehicle‐treated patients would achieve a ≥70% reduction in the dNNW ratio at visit 3. To secure a power of ≥90% in Pearson χ^2^ test, with a significance level of 5% (two‐sided), at least 30 patients per treatment group (total: 60) were required to complete visit 3.

Efficacy outcomes were evaluated in the full analysis set (FAS), which included all randomized patients who received at least one dose of topical treatment and had at least one postbaseline efficacy measurement. The safety analysis set included all randomized patients who received topical treatment and had safety data for the evaluation.

For the primary efficacy end point, the percentage of patients achieving a ≥70% reduction in the dNNW ratio on day 8 was calculated, along with the exact two‐sided 95% confidence intervals (CIs) for each group. Between‐group differences and 95% CIs were calculated via normal approximation using the estimated variance under the null hypothesis. *p*‐Values were calculated using Pearson χ^2^ test, with a two‐sided *α* < 0.05 considered significant. For the secondary end points, no adjustments were made for multiplicity of group or timepoint, and the significance level was uniformly set at 5% (two‐sided). Safety was determined using number (percentage). Statistical calculations were performed using SAS version 9.4 (SAS Institute Inc.).

## 3 RESULTS

### 3.1 Patient disposition and baseline characteristics

**FIGURE 1:**
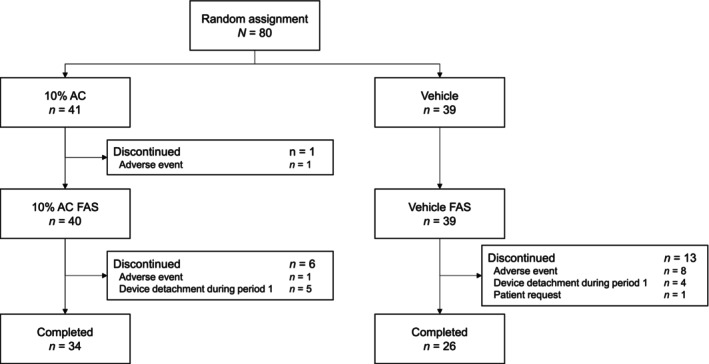
CONSORT (Consolidated Standards of Reporting Trials) diagram of patient disposition. AC, acetylcysteine; FAS, full analysis set.

The FAS included 79 patients (Figure 1). In the 10% AC and vehicle groups, respectively, the proportions of female patients were 80.0% (32 of 40) and 74.4% (29 of 39), and the mean ages were 50.8 years (standard deviation [SD], 12.3 years) and 53.0 years (SD, 11.7 years). In the 10% AC group, the mean dNNW ratio was 40.2% (SD, 6.7 [range, 20.5–49.0]) and the nail plate thickness was 1.4 mm (SD, 0.3 [range, 1.0–2.4]). The corresponding measurements in the vehicle group were 40.2% (SD, 7.1 [range, 21.0–50.0]) and 1.4 mm (SD, 0.3 [range, 1.0–2.3]).

### 3.2 Efficacy

**FIGURE 2:**
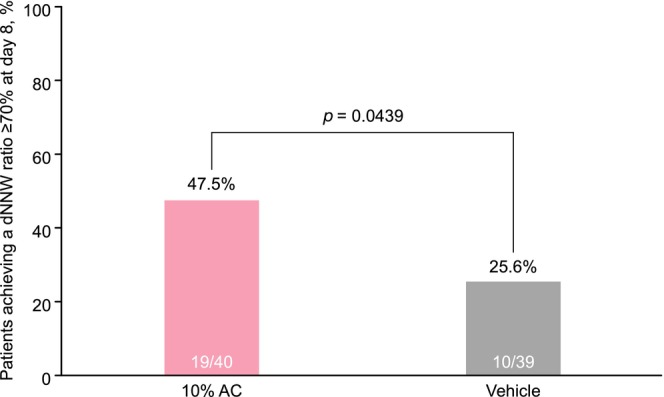
Rate of achievement of the primary end point of distal narrowed nail width (dNNW) ratio ≥70% at day 8 (full analysis set). AC, acetylcysteine.

The achievement rate of the dNNW ratio of ≥70% on day 8 was 47.5% in the 10% AC group and 25.6% in the vehicle group (Figure 2), with a between‐group difference of 21.9% (95% CI, 0.6–43.1; *p* = 0.0439).

The least‐squares means of the dNNW ratio on day 2 and day 8 (or discontinuation) were 68.4% (10% AC) and 66.1% (vehicle) on day 2 (between‐group difference: 2.3% [95% CI, −2.6 to 7.2]; *p* = 0.3478), and 69.3% (10% AC) and 61.7% (vehicle) on day 8 (difference: 7.6% [95% CI, 3.1–12.1]; *p* = 0.0012). The least‐squares mean values of the change in the dNNW ratio were 28.2% (10% AC) and 25.9% (vehicle) on day 2, and 29.0% (10% AC) and 21.5% (vehicle) on day 8.

**FIGURE 3:**
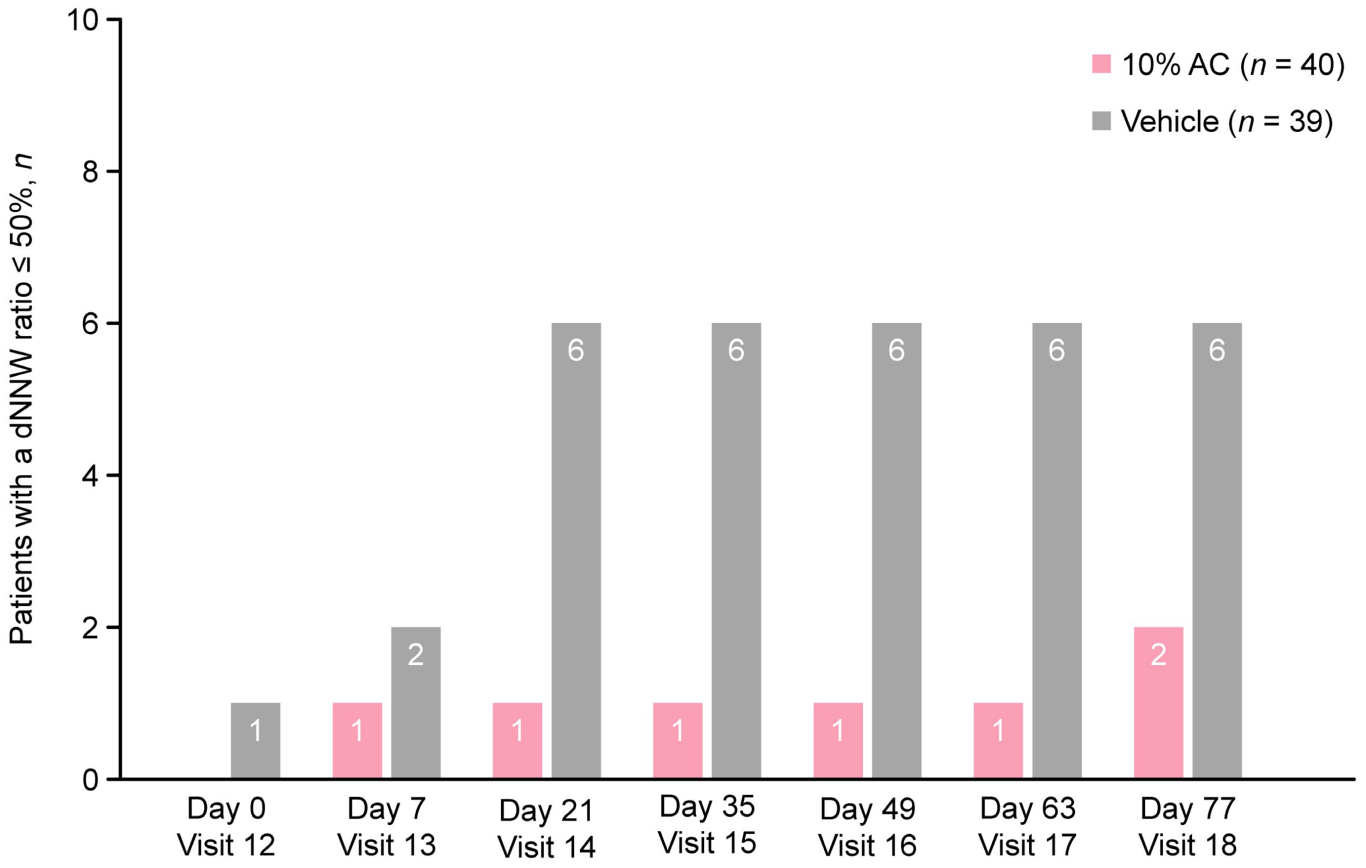
Days until the distal narrowed nail width (dNNW) ratio reached ≤50% during treatment period 4 (patients who transitioned directly from treatment periods 1 > 4 only). AC, acetylcysteine.

Overall, 28 patients transitioned directly from treatment periods 1 > 4 (18 in the 10% AC group and 10 in the vehicle group). Of these, 11.1% (two of 18) in the 10% AC group had a dNNW ratio of ≤50% by visit 18, and the other 16 patients maintained the correction effect. In the vehicle group, 60.0% (six of 10) of patients had a dNNW ratio of ≤50% by visit 18. The number of days until the dNNW ratio became ≤50% is shown in Figure 3.

**FIGURE 4:**
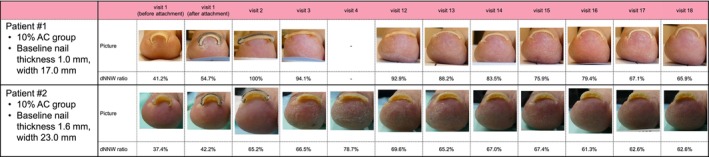
Individual patient morphologic outcomes over time. Patient #1 had thinner nails (<1.4 mm), achieved the primary end point (distal narrowed nail width [dNNW] ratio ≥ 70% on day 8) during treatment period 1, proceeded directly to period 4, and maintained a dNNW >50%. Patient #2 had thicker nails (≥1.4 mm) and did not achieve the primary end point in treatment period 1. This patient proceeded to treatment period 2, during which time a dNNW ratio ≥70% was achieved, and subsequently maintained a dNNW >50% during treatment period 4. AC, acetylcysteine.

When evaluated by nail thickness <1.4 mm, the mean change in the dNNW ratio on day 8 was 33.5% (10% AC) and 22.7% (vehicle). A corrective effect was also observed with thicker nails (≥1.4 mm; Figure 4); corresponding changes were 27.5% (10% AC) and 21.4% (vehicle).

### 3.3 Safety

The incidence of AEs was 37.5% (15 of 40 patients) in the 10% AC group and 56.4% (22 of 39) in the vehicle group (Table 1). Most AEs were mild; one patient in each treatment group had an AE of moderate severity (10% AC, tendonitis; vehicle, tenosynovitis), and one patient (10% AC) had a severe AE (breast cancer). There were no AEs resulting in death.

**TABLE 1:** Summary of safety outcomes (safety analysis set).

	All AEs		Drug‐related AE		Device‐related AE	
	10% AC	Vehicle	10% AC	Vehicle	10% AC	Vehicle
	(*n* = 40)	(*n* = 39)	(*n* = 40)	(*n* = 39)	(*n* = 40)	(*n* = 39)
AE, *n* (%)	15 (37.5)	22 (56.4)	0	6 (15.4)	6 (15.0)	15 (38.5)
Mild	14 (35.0)	22 (56.4)	0	6 (15.4)	6 (15.0)	15 (38.5)
Moderate	1 (2.5)	1 (2.6)	0	0	0	0
Severe	1 (2.5)	0	0	0	0	0
Serious AE, *n* (%)	1 (2.5)^a^	0	0	0	0	0
AESI, *n* (%)	2 (5.0)	10 (25.6)	0	2 (5.1)	0	9 (23.1)
Inflammation^b^	2 (5.0)	1 (2.6)	0	1 (2.6)	0	1 (2.6)
AE requiring device removal	0	9 (23.1)	0	1 (2.6)	0	8 (20.5)
Other AE occurring at the nail, *n* (%)^c^						
Nail damage/breakage	2 (5.0)	9 (23.1)	0	1 (2.6)	0	8 (20.5)
Skin pain	1 (2.5)	1 (2.6)	0	1 (2.6)	0	1 (2.6)
Onycholysis	0	1 (2.6)	0	0	0	0
Damage at the drug application site	2 (5.0)	2 (5.1)	0	1 (2.6)	2 (5.0)	2 (5.1)
Cracks at the drug application site	3 (7.5)	0	0	0	1 (2.5)	0
Pain at the drug application site	2 (5.0)	0	0	0	2 (5.0)	0
Pain caused by the medical device	1 (2.5)	1 (2.6)	0	0	1 (2.5)	1 (2.6)
Traumatic hematoma	0	1 (2.6)	0	0	0	0

*Note*: Adverse events (AEs) were categorized using Medical Dictionary for Regulatory Activities/Japanese edition (MedDRA/J) version 23.0.

Abbreviations: AC, acetylcysteine gel; AESI, adverse event of special interest.

^a^ Breast cancer.

^b^ Inflammation around the nail to which the study drug was applied (i.e. erythema, edema). All three cases were categorized as paronychia (MedDRA/J System Organ Class [SOC] ‘infection and parasitism’) and none were categorized as contact dermatitis (SOC ‘skin and subcutaneous tissue injury’).

^c^ On the nails or on the skin around the site of investigational drug application.

The most frequently reported AE was nail damage, occurring in 5.0% (two of 40 patients) in the 10% AC group and 23.1% (nine of 39) in the vehicle group (Table S1). In the 10% AC group, both cases were mild, and were found to be due to nail bumping or scratching; it was judged that there was no causal relationship with either the study drug or the device. In the vehicle group, all nine cases of nail damage were mild in severity (see Figure S4 for examples). One case was judged to be unrelated to either vehicle or device but required device removal. Eight cases were judged to be device‐related (two occurred during the administration period and six during the device‐wearing period after removal of the vehicle) and seven of these resulted in device removal.

There were no drug‐related AEs in the 10% AC group. In the vehicle group, 15.4% (six of 39 patients) had drug‐related AEs. Device‐related AEs occurred in 15.0% (six of 40) in the 10% AC group and 38.5% (15 of 39) in the vehicle group; the most frequent were nail damage (vehicle group only, 20.5% [eight of 39]; described above) and damage at the drug application site (10% AC group, 5.0% [two of 40]; vehicle group, 5.1% [two of 39]).

The AESI of inflammation around the nail occurred in 5.0% (two of 40 patients) in the 10% AC group (not judged to be drug‐ or device‐related) and in 2.6% (one of 39) in the vehicle group (judged to be both drug‐ and device‐related). All three cases were categorized as paronychia, and no cases of contact dermatitis due to AC were reported. Nine patients in the vehicle group required the overcurvature‐correcting device to be removed (one due to a drug‐related AE [application site injury] and eight due to device‐related AEs [nail breakage]), compared with no patients in the 10% AC group.

## 4 DISCUSSION

In this phase 3 study, a 24‐h application of 10% AC gel was sufficient to soften the hallux nail, allowing earlier correction of the pincer nail compared with an overcurvature‐correcting device alone, which is consistent with our phase 1/2 study.^12^

The primary end point was met, with a significantly greater achievement of the dNNW ratio of ≥70% in the 10% AC group versus vehicle on day 8. Secondary end points also showed a greater tendency towards improvement in the 10% AC group versus vehicle, confirming the earlier therapeutic effect of combining 10% AC gel with the overcurvature‐correcting device. Data from treatment period 4 after device removal demonstrated that the nail correction effect was maintained over time in the majority of AC‐treated patients.

No AC‐related AEs were reported, and no AEs requiring device removal occurred in the 10% AC group. Nail damage was more frequent in the vehicle group; we consider it likely that the softening (keratin‐destabilizing) effect of AC gel^10^, ^11^ resulted in a lower likelihood of subsequent damage. In contrast, the nails of vehicle‐treated patients may have become temporarily softened due to application of a liquid and weakening of the hydrogen bonds,^14^ but returned to hardness and transverse overcurvature following drying.

The softening effect of AC gel, which changes the fine structure of the nail, is also the main factor underlying its usefulness in pincer nail correction. We believe that AC gel softens nails by breaking the disulfide bonds of keratin during the 24‐h application period. Following AC gel removal, the subsequent recombination of the disulfide bonds while the nail shape is being corrected by the device helps to maintain the correction, resulting in enduring improvements once the device is removed. Although noninvasive superelastic wires^15^ and formable acrylics^16^ have been introduced as potential pincer nail treatments, it can still take several months to a year to achieve a good corrective effect with a device alone.^7^ Similarly, use of a plate takes more than 6 months, and recurrence can occur in less time than was required for the initial correction.^2^ The use of nail‐softening agents to enhance the corrective effects of an overcurvature‐correcting device has been previously reported,^7^ and application of urea, which has a water‐retaining effect, is frequently used to soften the nail plate in pincer nails and other conditions.^17^, ^18^, ^19^ However, conservative treatment of pincer nail with uric acid ointment also requires a considerable time duration.^17^ In contrast, our study suggests that a single application of 10% AC gel, in combination with attachment of an overcurvature‐correcting device for 7 days, can produce an early and sustained therapeutic effect.

Currently, either conservative or surgical procedures can be performed to treat pincer nail,^1^ and although there is no consensus on a standard treatment pathway, conservative treatment should generally be considered in the first instance. Although the recurrence rate following surgery may be lower than that following conservative treatment, invasive surgery requires specialist skills and is associated with several risks including significant levels of pain, impaired appearance of the affected area, secondary infection, wound necrosis, and sensory impairment.^20^, ^21^ Patient characteristics (such as age, presence of uncontrolled diabetes, or arterial insufficiency) may also preclude surgical intervention.^22^ For many patients, conservative treatment using an overcurvature correction device is able to provide a permanent solution without the need for more aggressive intervention,^23^, ^24^ although repeat procedures may be necessary to prevent recurrence and achieve long‐term success. However, while discrete overcurvature correction devices can allow treatment without overly impeding daily life, the often lengthy durations required to achieve correction may be daunting for patients. Thus, our technique, which is noninvasive and requires no specialist skills, and also reduces the time needed for nail improvements by combining chemical softening with a device, is likely to be of interest to both physicians and patients. Study limitations include that only patients with pincer nails ≥1.0 mm in thickness were eligible, and we cannot extrapolate our findings to patients with thin or brittle nails. Success rates for patients with thicker and harder nails than those evaluated herein also remain to be determined. Only one type of device was tested, although other overcurvature‐correcting devices may be equally effective due to a similar physical mechanism of action. Finally, the study duration was not sufficient to fully examine the possibility of recurrence, and further long‐term investigation is warranted.

In summary, combination therapy with AC gel and an overcurvature‐correcting device rapidly improves pincer nails, with a low treatment burden for patients, and can maintain the shape of the nail for a long time following a single application of AC gel. Thinner nails tended to improve more rapidly, but therapeutic effects could be obtained even with thicker nails. We believe that this combination treatment could be a useful option for the treatment of pincer nails.

## FUNDING INFORMATION

Maruho Co., Ltd.

## CONFLICT OF INTEREST STATEMENT

M. Saito has received payment for speaking, manuscript preparation, and expert testimony from Maruho Co., Ltd. A. Ikoma, A. Fujikawa, and K. Tanaka are employees of Maruho Co., Ltd.

## Supporting information


Appendix S1

## Data Availability

The data that support the findings of this study are available from the corresponding author upon reasonable request.
