# Supplementary material for: Improvements in pincer nail deformity occur earlier and last longer when acetylcysteine gel is added to an overcurvature‐correcting device: Results of a phase 3, multicenter, randomized, vehicle‐controlled, investigator‐blinded study
Source: J Dermatol. 2023 Nov 27;51(1):23–9. doi: 10.1111/1346-8138.17010 (PMC11483967; doi:10.1111/1346-8138.17010)
Supplement: Supplementary file 1 — Appendix S1 [file JDE-51--s001.docx]

# Supplementary Materials

### **Text S1.** List of study sites

The six Japanese facilities (and investigators) involved in the study were as follows:

1. Medical Corporation Shinanokai Shinanozaka Clinic (Masataka Saito);

2. Saiseikai Kawaguchi General Hospital (Kaoru Takayama);

3. Hiroo Dermatology Clinic (Tatsuya Izumi);

4. Medical Corporation Chiseikai Tokyo Center Clinic (Mana Hirayama);

5. Medical Corporation Yuushikai Shinnakano Dermatology Clinic (Kyoko Oyama);

6. Kanda Suzuki Skin Clinic (Hirotake Suzuki).

**Figure S1.** Calculation of the distal narrowed nail width (dNNW) ratio


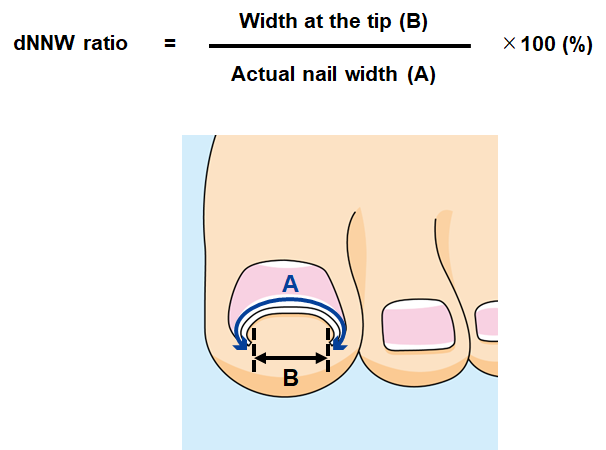


**Figure S2.** Schematic of the study treatment periods


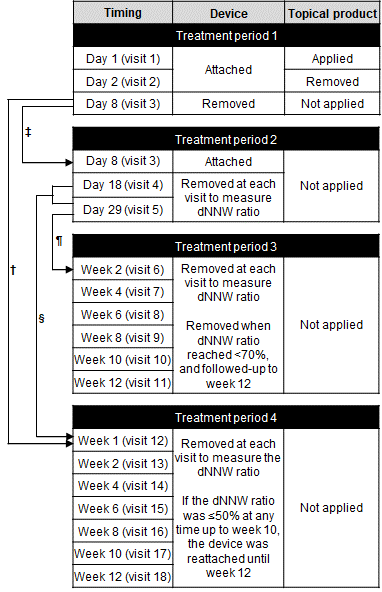


dNNW, distal narrowed nail width.

^†^Patients with a dNNW ratio ≥70% on day 8 moved directly to treatment period 4.
^‡^Those with a dNNW ratio <70% on day 8 moved to treatment period 2.

^§^Patients with a ratio of ≥70% on day 18 or day 29 transitioned to treatment period 4.
^¶^Patients who continued to have a ratio of <70% on day 29 continued with device attachment and transitioned to treatment period 3.

**Figure S3.** Instructions for study treatment application


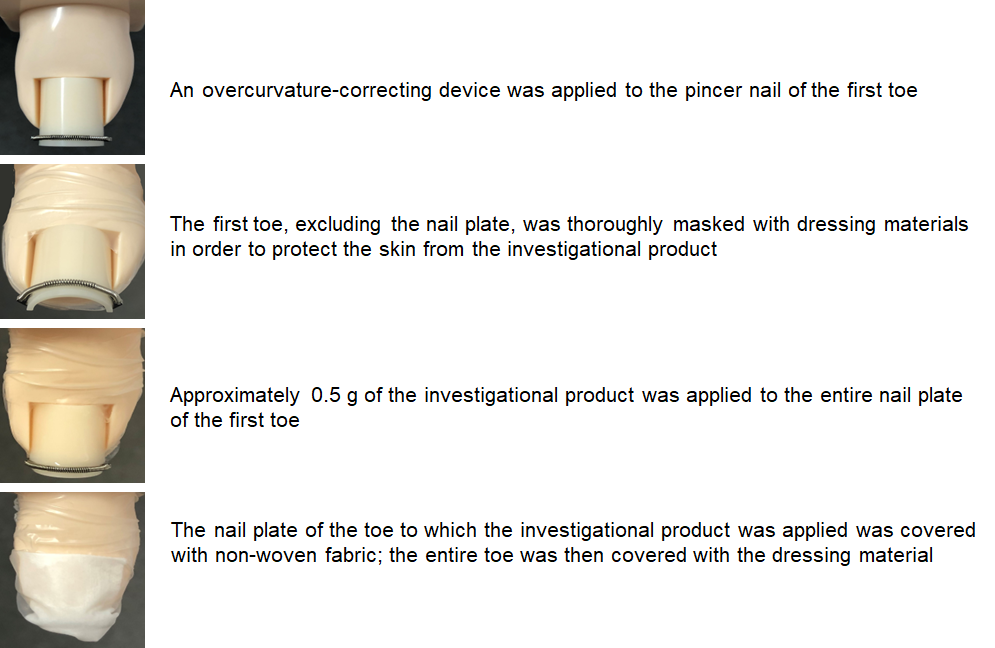


# FIGURE S4. Device-associated nail damage in the vehicle group.


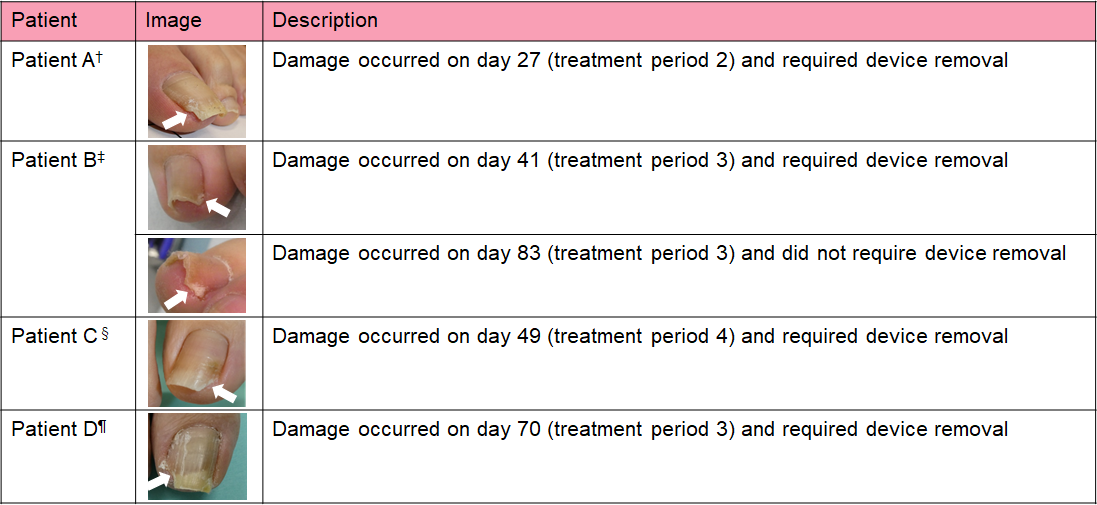


^†,‡,§,¶^Symbols refer to patients described in **Table S1**.

**TABLE S1.** Summary of nail damage in the 10% AC and vehicle groups.

| **Group** | **Time of emergence** | **Severity** | **Drug-related** | **Device-related** | **Removal of device** | **Nail thickness** | **Causal basis** |
| --- | --- | --- | --- | --- | --- | --- | --- |
| 10％ AC | Day 63 (period 4) | Mild | No | No | N/A | 1.0 mm | The nail was hit on the stairs, causing breakage |
|  | Day 73 (period 4) | Mild | No | No | N/A | 1.4 mm | The nail got caught and chipped |
| Vehicle | Day 2 (period 1) | Mild | No | No | Yes | 1.0 mm | Damage thought to result from a strong external (clothing-related) force |
|  | Day 8 (period 1) | Mild | Yes | Yes | No | 1.0 mm | Damage thought to result from device-related force following softening |
|  | Day 8 (period 1) | Mild | No | Yes | Yes | 1.2 mm | Damage thought to result from the device |
|  | Day 27  (period 2)^†^ | Mild | No | Yes | Yes | 1.4 mm | Damage thought to result from device-related force as the nail grew and rehardened; no drug relationship due to time elapsed since application and removal |
|  | Day 39 (period 4) | Mild | No | Yes | Yes | 1.2 mm | No drug relationship due to time elapsed since application and removal |
|  | Day 41 (period 3)^‡^  Day 83 (period 3)^‡^ | Mild  Mild | No  No | Yes  Yes | Yes  No | 1.5 mm | No drug relationship due to time elapsed since application and removal |
|  | Day 44 (period 3) | Mild | No | Yes | Yes | 1.3 mm | No drug relationship due to time elapsed since application and removal |
|  | Day 49  (period 4)^§^ | Mild | No | Yes | Yes | 1.1 mm | Damage thought to result from the device |
|  | Day 70 (period 3)^¶^ | Mild | No | Yes | Yes | 1.9 mm | Damage thought to result from device attachment and detachment in a short time period |

N/A, not applicable.

Symbols refer to Patients ^†^A, ^‡^B, ^§^C, and ^¶^D in **Figure S4**.
